# Medium and long-term results of gastric banding: outcomes from a large private clinic in UK

**DOI:** 10.1186/s40608-018-0189-1

**Published:** 2018-04-12

**Authors:** Leeying Giet, Julia Baker, Franco Favretti, Gianni Segato, Paul Super, Rishi Singhal, David Ashton

**Affiliations:** 1 Heart of England NHS Foundation Trust, Birmingham, UK; 2 Healthier Weight Centres, 11 Highfield Road, B15 3DU, Birmingham, UK; 3 Birmingham, England

**Keywords:** Bariatric surgery, Laparoscopic adjustable gastric band, Obesity, Weight loss, Private

## Abstract

**Background:**

Laparoscopic adjustable gastric band (LAGB) has been an established bariatric procedure for the last three decades and was, for many years, the first-choice procedure for the treatment of chronic obesity. However, more recently, the popularity of the LAGB has been in sharp decline and has been replaced by other procedures such as the Roux-En-Y gastric bypass and sleeve gastrectomy. A key driver in this decline has been the high revision and early explanation rates reported in some studies.

**Methods:**

This was a retrospective study of 2246 patients who underwent LAGB at a large private clinic in the UK between June 2004 and October 2014.

**Results:**

2246 patients were included in the study; 1945 (84.6%) were women. All patients were followed up for 2 years following their procedure and subsequent follow up was at the discretion of patients. Mean follow up duration was 43.7 +/− 29.3 months. Operative mortality was zero and there were no in-hospital returns to theatre. Mean preoperative weight and body mass index (BMI) were 111.2 ± 22.1 kg and 39.9 ± 6.7 kg/m^2^ respectively. Mean excess % BMI loss at 1-, 2-, 5- and 8-years of follow-up was 43.1 ± 25.4, 47.9 ± 31.9, 52.4 ± 41.7 and 57.1% ± 28.6 respectively. There was no significant difference in mean excess % BMI loss between those < 50 or ≥ 50 years old (*p* value = 0.23) or between patients with an initial BMI of < or ≥ 50 kg/m^2^ (p value = 0.65). Complications over nine years occurred in 130 (5.8%) patients and included: 39 (1.7%) slippage or pouch dilatation, 2 (0.04%) erosions and 76 (3.4%) problems related to the access port or LAGB tubing. The overall re-operation rate for LAGB complications was 4.2% over 9 years with a LAGB explantation rate of 1.5%. 39 LAGBs were converted to a sleeve or gastric bypass procedure, 11 of these due to complications.

**Conclusion:**

This is the first study to report on LAGB outcomes from a private clinic in the UK. LAGB is a safe procedure, which delivers significant and durable weight loss with acceptable complications rates and low re-operation rate.

## Background

The first laparoscopic implantations of the adjustable gastric band (LAGB) were performed in the last decade of the last century [1]. In the almost 25 years which have elapsed since then, scores of studies have confirmed the important role for bariatric surgery in the management of patients with obesity [2]. At the time of introduction of the LAGB, open vertical banded gastroplasty (VBG) and Roux-en-Y gastric bypass (RYGB) were the predominant procedures and were associated with morbidity and mortality. The advent of LAGB offered a safe and effective alternative, which delivered durable weight loss, with major improvements in obesity-associated co-morbidities [3].

As experience with the LAGB grew, it quickly became the most commonly performed procedure in the UK, especially in the private sector. This popularity was driven by strong celebrity endorsement and extensive press and media coverage. Moreover, the fact that the LAGB could be safely implanted as a day-case procedure, added to a growing perception among the public that the LAGB offered a low-risk, quick and simple solution for those struggling to lose weight [4].

During the last 5 years there has been a decline in the use of LAGB due to multiple reports showing unacceptably high complication and early explantation rates [5–10]. In a report published in 2009, 56.1% of bariatric procedures worldwide were LAGB [11]. Since then, the use of RYGB and laparoscopic sleeve gastrectomy (LSG) has increased, with LSG becoming the main bariatric surgical procedure globally [12].

In the UK, the National Bariatric Surgery Register (NBSR) includes details regarding bariatric surgery, primarily from National Health Service (NHS) hospitals [13]. There has been a reduction in the number of providers offering LAGB in the UK over the last 5 years and NHS centres offering this procedure outside the By-Band study are scant [14]. However, LAGB remains a common procedure in the private sector in the UK due to patient’s choice and data for these procedures are not included in the NBSR. Given that these procedures are being performed outside of the NHS, with the NHS often dealing with their complications, it is essential that the outcomes of LAGB in the private sector are examined.

In this study we aimed to examine the weight loss outcomes and complication rates in a large cohort of LAGB patients from a single, private provider in the UK. Although only LAGB outcomes were examined, this clinic offers the entire breath of bariatric surgery including intra gastric balloon, laparoscopic sleeve gastrectomy, laparoscopic Roux-en-Y Gastric bypass, revisional bariatric surgery and endoscopic sleeve gastroplasty.

## Methods

A retrospective cohort study of all cases of LAGB by a large, private weight management clinic in the UK between June 2004 and October 2014 were included. All patients benefited from a 2 year aftercare programme as part of their LAGB package. This included unlimited adjustments, access to a dietetic team and surgical consults if required. Fluoroscopic evaluations and any necessary management of complications was also included. The aftercare package encouraged patients to maintain regular contact with the clinic. Beyond the aftercare package, the clinic operated an open door policy through which patients could access clinic reviews to discuss their weight loss and possible complications, as and when necessary.

Data was collected from computer records and from clinic appointments. Patients who were beyond 36 months following their primary procedure with no recent weight loss data were initially contacted with e-mail questionnaires. Those that did not respond were subsequently contacted via telephone consultations. Only patients who had given explicit permission to be contacted via telephone consultations pre-operatively were contacted in this way. All contacts were made by the clinical team who provided care for these patients. Data was thus available for the first two years as a result of direct follow up and for another two years as a result of retrospective contact via email and telephone. Data collection included basic demographics, date of surgery, pre-operative weight, height, body mass index (BMI), post-operative complications and most recent weight. Collection and reporting of co-morbidity data was not performed.

Weight loss was calculated for patients who were more than 12 months from their initial procedure and have been reported in percentage excess BMI loss (% excess BMI loss) [15]. Patients who required further revisions/ reoperations following their initial procedure were excluded from our weight loss analysis but reported separately.

Patients were admitted on the day of their surgery. The procedure was performed laparoscopically with either a four or five port technique or a single incision (SILS). The pars flaccida technique was employed in all cases for LAGB insertion. Gastropexy/ tunnelling sutures were used in all cases to secure the LAGB. The number and the placement of sutures depended on surgeon’s personal preference. Ports were routinely fixed to the anterior abdominal wall (type of fixation technique varied according to individual surgeon preference). A variety of saline filled LAGBs were used including Swedish Adjustable Gastric Band, LAP-BAND AP®, A.M.I® and Bioring®.

Patients were routinely discharged on the same day with a prescription of low molecular weight heparin (LMWH) for 7 days and TED stockings. A build up to a normal textured diet from a liquid to soft diet was usually achieved over a period of six weeks. Clinical LAGB adjustments were performed by either a consultant or nurse specialist in clinic. Fluoroscopy was utilised in cases of difficult access or suspected complications. As part of their package, all patients were followed up for a period of at least two years following their procedure. Following this, patients were reviewed if they had paid for extra follow up or LAGB adjustments. Follow up consisted of an initial six weeks, three months and six months follow up, followed by six monthly follow up until two years by a team consisting of bariatric nurses, dieticians and surgeons. Open access was available to all patients, including outside of their paid contract.

Statistical analysis was performed using the Statistical Package for Social Sciences, version 17 (SPSS, Chicago, IL). Data was reported in frequencies and mean ± standard deviations or median (IQR) depending on data distribution. Comparison between baseline and follow up weight was performed using the paired t test or the Wilcoxon signed-rank test depending on data distribution. Data distribution was determined using the Shapiro Wilk test. *P* value < 0.05 were considered significant unless stated otherwise.

## Results

### Basic demographics

**Fig. 1:**
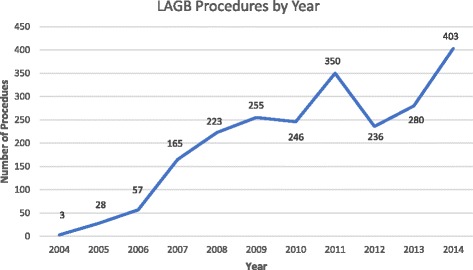
LABG procedures by year

Between June 2004 and October 2014, 2246 patients underwent primary implantation of a LAGB. Of the LAGBs implanted, 81% were performed by three experienced consultant bariatric surgeons. The mean age of these patients was 45.6 ± 11.5 years. 84.6% (1945) of patients were females. The mean baseline weight was 110.6 ± 22 Kg and the baseline BMI was 39.7 ± 6.6 Kg/m^2^. The baseline BMI range was 28.5–73.4 Kg/m^2^ (median 38.6 Kg/m^2^). Since 2004, there has been an increase in the number of LAGBs performed by this clinic (Fig. 1). However, of late, other bariatric procedures are increasing in popularity, with LAGBs now only constituting 56% of all procedures performed by this clinic. The mean follow up was 43.7 +/− 29.3 months.

### Data reporting

**Fig. 2:**
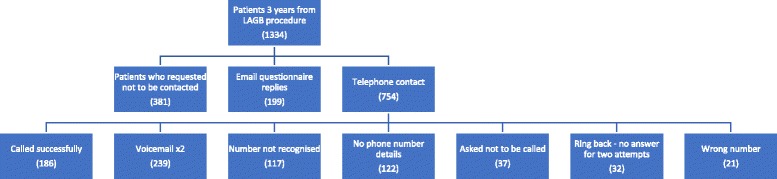
Outcomes from telephone consultations

A total of 1334 patients were beyond 36 months following their procedure. Patients for whom we had no weight loss data from 36 months onwards were contacted via email or phone depending on the patients indicated preference. The outcome of this has been depicted in Fig. 2.

### Weight loss

A total of 1640 patients were over 12 months from their initial procedure and had not reported any complications. 84.3% patients were female. Their mean preoperative weight and BMI were 111.2 ± 22.1 kg and 39.9 ± 6.7 kg/m^2^ respectively. Mean excess BMI loss over 108 months from the initial procedure is reported in Table 1. 80.48% of patients had complete follow-up 24 months post operatively.

**Table 1:** Mean % excess BMI loss for all patients

Time period (months)	Mean % excess BMI loss	Standard deviation	Follow up(%)	Follow up method
1.5	21.9	13.2		
3	26.4	16.1		
6	33.9	19.7	96.67	Follow up at Healthier Weight clinic
12	43.1	25.4	78.08	
18	45.4	30.1	56.98	
24	47.9	31.9	80.48	
36	47.4	34.1	23.60	Email and telephone based follow up
48	49.4	35.9	19.58	
60	52.4	41.7	Follow up not calculated. Open door policy enabling patient initiated follow up	
72	52.3	33.9		
84	50.8	34.9		
96	57.1	28.6		
108	54.8	47.1		

### Age and weight loss

**Fig. 3:**
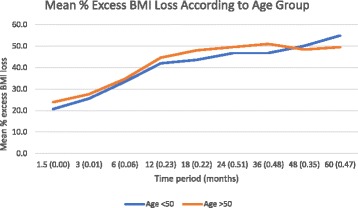
Mean % excess BMI loss according to age group with statistical significance at each time point

Patient were divided into two groups depending on age (< 50 (1305 patients) and ≥ 50 years (762 patients) [16]). Mean excess % BMI loss figures were compared between these two groups. There was no statistically significant difference in their baseline mean BMIs (Age < 50–40.1 +/− 6.8 vs. 39.5 +/− 6.6, *p* = 0.05). Between the groups, mean excess % BMI loss was initially better in patients who were over the age of 50 for up to three months following their procedure (Fig. 3). However, there was no statistically significant difference following this.

### BMI and weight loss

**Fig. 4:**
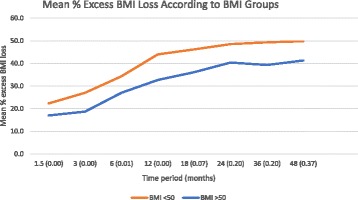
Mean % excess BMI loss according to BMI groups with statistical significance at each time point

Patients were divided into two groups of BMI (BMI < 50 vs BMI ≥50) for analysis. There were 1504 patients with a BMI < 50 and 134 patients with a BMI ≥50 k/m^2^. The mean baseline BMIs were 38.5 ± 5.0 and 54.9 ± 4.6 kg/m^2^ respectively (*p* = 0.02). Statistically better mean excess % BMI loss was achieved for patients with a baseline BMI of < 50 kg/m^2^ for the first 12 months (*p* < 0.05, Fig. 4). However, this difference lost statistical significance after this.

### Follow up

For weight loss reporting, follow up has been calculated for the first 2 years as a percentage of the patients eligible for follow up at that time point. Follow up was 96.67%, 78.08%, 56.98% and 80.48% at 6, 12, 18 and 24 months respectively, as a result of direct follow up. Complications were reported over a period of 98 months.

### Complications

A total of 2246 patients had a saline filled LAGB implanted, of which a total of 130 complications were identified (5.8%) and detailed in Table 2. Yearly complication rates are described in Table 3.

**Table 2:** LAGB complications. Relative distribution of LAGB complications (*n* = 2246)

Complication	Number	Percentage (%)	Reoperation rate (%) and number
Mortality	0	0	0 (*n* = 0)
Tubing complications	24	1.0	0.7 (*n* = 16)
Port complications	45	2.0	1.8 (*n* = 40)
Slippage	32	1.4	1.4 (*n* = 31)
Pouch dilatation	7	0.3	0.1 (n = 2)
Erosion	2	0.1	0.04 (n = 1)
Infection	7	0.3	0.3 (*n* = 7)
Pseudo-capsule	12	0.5	0.3 (n = 7)
Foreign body left in situ	1	0.04	0.04 (n = 1)
Total	130	5.78	4.6 (*n* = 105)

**Table 3:** Complications by year

Year	No. of complications by year	Yearly complication rate (%)
1	29	1.56
2	23	1.44
3	12	3.95
4	8	4.26
5	6	Complication rates not calculated. There was an open door policy enabling patient initiated follow up.
6	1	
7	3	
8	1	
9	0	

Of the major complications, 39 (1.7%) experienced a slippage or pouch dilatation which required 33 re-operations (1.5% re-operations for slippages); removal - 12; replacement - 13; repositioning - 4 and conversion to Roux-en-Y-gastric bypass (RYGB) or SG - 4. It is to be noted that only 9 slippages were diagnosed within 2 years of follow up (1.3%).

2 patients had erosions with one resulting in a conversion to RYGB (0.04%). The second patient was lost to follow-up.

76 (3.4%) patients had problems related to the tubing or port including infections with 63 patients undergoing reoperations; 7 LAGB or port removals, 49 LAGB or port replacements, 6 conversions to RYGB or SG and 1 tubing repair.

One further patient had a foreign body left in situ from their primary operation and which required a reoperation for removal. All complications have been listed in Table 2.

### Other reoperations (not due to complications)

#### Conversions

28 (1.2%) patients underwent a conversion from their LAGB to either RYGB or SG. This was due to inadequate weight loss in 9 patients (between 21 and 112 months after the initial procedure) and individual choice in 12 (between 5 and 81 months). There was no data available to account for conversion in the remaining 6 (12–68 months).

#### LAGB removals, replacements or repositionings

A further 29 (1.3%) patients had their LAGBs removed, replaced or repositioned. Of these, 16 were removed for no obvious reason, 7 due to patient choice and one due to a fracture of the LAGB tubing secondary to a road-traffic accident. Three LAGBs were replaced and two were repositioned for reasons unknown.

## Discussion

The overall popularity of LAGB has been declining. Australia, UK, Israel, Canada and Belgium are the few countries with a reasonable market share for LAGB amongst the bariatric procedures. This study is thus unique since it aims to present data to a sceptical bariatric society.

Operative mortality in this cohort was zero and there were no in-hospital returns to theatre, confirming that the LAGB is an extremely safe procedure. The study has the advantage of a large cohort, but the obvious disadvantage that – despite strenuous efforts to contact patients - the follow-up rates could have been better.

The observed weight loss in our study is consistent with many previous reports and the pattern of weight loss was typical of the LAGB – a gradual rise followed by a stable plateau at around 50% excess BMI loss [17, 18]. This contrasts with procedures such as SG and RYGB, where the pattern tends to be one of substantial initial weight loss, followed by gradual weight regain [16, 19]. In fact, a number of studies have shown that the initial advantages of procedures such as SG and RYGB are attenuated at ≥5 years and that beyond this, there is little difference in outcomes [20].

Age and BMI as predictors of weight loss outcomes after bariatric procedures has been reported in several studies. In general, older patients (> 50 years) seem to do as well as younger age-groups and this observation has been confirmed in the present study^16,21.^ Our finding that weight loss in the first 12-months was greater in those with a pre-operative BMI < 50 kg/m^2^ than those in the BMI ≥50 kg/m^2^ group, but that there was no difference for years 2-,3- and 4, is precisely the same observation made by Dixon and O’Brien [21]. Others have also found no significant difference in weight loss between the obese and the super-obese [22].

Complications of LAGB are widely reported in the literature. LAGB slippage has a highly variable incidence of anywhere between 1 and 22% [23–27]. O’Brien and Dixon reported a LAGB slip rate of less than 5% and a recently reported review of LAGBs from a UK-based facility, reported a rate of 3.1% in a single-centre cohort of 719 patients [28, 29]. In a five-year follow up of 2815 LAGB patients, Coburn et al. reported a slippage rate of 4.2% [4]. Thus, our overall slippage rate of 1.4% in the present study is at the very lowest end of the range. It is important to note that all our LAGBs were implanted using a *pars flaccida* approach, which is known to be associated with lower rates of complications than the perigastric technique. For example, Ponce et al. reported a 20.5% slippage rate with the perigastric approach, which decreased to 1.4% after adopting the pars flaccida technique and O’Brien et al. have shown the risk of anterior slippage to be almost four times higher for the perigastric approach compared with the *pars flaccida* [30, 31].

Erosion (migration) is another important, though uncommon, complication of the LAGB. The reported incidence varies between 0.5–3.3% and requires removal of the device, which can usually be achieved endoscopically [2, 4, 32]. In the current study erosion was a rare complication of LAGB, with an incidence of just 0.1%.

Port and tubing complications, including leakages and infections, were observed in 76 (3.4%) patients. Once again, there is a wide variation in the literature with rates varying between 1.2–24%, often depending on the length of follow-up [4, 33]. Most complications of this kind are eminently correctable, usually by replacing or re-positioning the access port or, occasionally, replacing the entire LAGB.

A key factor in the decline in LAGB has been the high revision and early removal rates of up to 60% in some publications [5–10]. However, very much lower revision rates have also been reported. For example, Coburn et al., in a 5-year follow-up of 2815 LAGB patients reported complications in 8.5% with an explantation rate of just 1.2% [4]. In a 12-year follow-up of 1791 patients, Favretti et al. report a re-operation rate of 5.9% and LAGB removal in 3.7% [22]. Similarly, in a 5-year follow-up of 442 patients, Ray and Ray reported % EWL of 60% at 5-years, with a slippage and erosion rate of 2% and 0.4% respectively and an explantation rate of 1.8% [34]. In the present study, we found a total 105 (4.6%) of patients requiring re-operation for LAGB complications, necessitating removal in 35 (1.5%). However, these results need to be tempered with an 80.48% follow up of those eligible at 2 years. We also need to acknowledge that most patients were not in a paid follow up programme after 2 years and only presented to us if they wanted to manage their complications in the private sector.

Thus, our results lend strong support to the currently unfashionable view that, when correctly applied and managed, the LAGB is a safe, effective and durable short-stay procedure which can be safely revised and is well tolerated by patients^37.^ We of course concede that the outcomes of LAGB are variable both in terms of revision and explanation rates. An appraisal of the multicentre Realize study [26] and the French study by Lazzati *et al* [35] which involved almost 53,000 patients confirmed that outcomes were highly dependent on the experience of the teams. Moreover, band salvage was more likely if the patient had presented to the original implanting hospital.

Based on these findings, we believe that sub-optimal outcomes for the LAGB are primarily a function of low volume surgeons or facilities and an inadequate LAGB aftercare programme. The issue of sub-optimal follow-up is of relevance in the National Health Service (NHS) because of chronic funding problems. The LAGB is perceived to be a resource-intensive intervention which, because of the requirement for regular adjustments and clinic visits, places an undue burden on an already limited healthcare funding. The fact is that LAGB requires a long-term commitment to the patient and whilst this is obviously true for all bariatric patients, it is an *indispensable* requirement for success with the LAGB. In circumstances where they cannot provide the requisite level of support, many NHS surgeons will feel that this is a commitment they simply cannot make and will opt for alternative procedures.

## Conclusions

This study like any other has its own flaws and data should only be extrapolated with care. It is arguable that patients who remained in follow up were the patients who were satisfied with the clinic and had good outcomes. It is thus conceivable that patients with poor outcomes or those who developed complications presented to other clinics or hospitals and avoided contact with us. That said, all reasonable attempts were made to contact patients wherever possible. The majority of the procedures in this study were performed by three very experienced surgeons and LAGB adjustments were performed by a dedicated and motivated team. Most importantly, these were self-paying patients and as such, their motivation to succeed is likely to be greater than NHS patients. Thus, the results may not be completely extrapolated to NHS practice. Similarly, although the range of BMIs operated upon at our clinic was diverse, the co-morbidities for these patients are likely to be less severe when compared with their NHS counterparts.

Overall, we feel that it is likely that most LAGBs implanted in the UK will continue to be in the private sector. Assuming that this is the case and that the LAGB survives the current fashion for more aggressive procedures, our results should be reassuring to both patients and regulatory authorities alike. As far as the authors are aware, this is the first such report from the private sector in the UK.

## Abbreviations

BMI: Body Mass Index
EWL: Excess Weight Loss
LAGB: Laparoscopic Adjustable Gastric Band
LMWH: Low Molecular Weight Heparin
LSG: Laparoscopic Sleeve Gastrectomy
NBSR: National Bariatric Surgery Registry
NHS: National Health Service
RYGB: Roux-en-Y Gastric Bypass
SILS: Single Incision Laparoscopic Surgery
VBG: Vertical Banded Gastroplasty
